# Genome Sequence of the Urethral Catheter Isolate Pseudomonas aeruginosa MH19

**DOI:** 10.1128/genomeA.00115-15

**Published:** 2015-03-12

**Authors:** Frank-Jörg Vorhölter, Petra Tielen, Daniel Wibberg, Maike Narten, Max Schobert, Reinhilde Tüpker, Jochen Blom, Sarah Schatschneider, Anika Winkler, Andreas Albersmeier, Alexander Goesmann, Alfred Pühler, Dieter Jahn

**Affiliations:** a Institute of Microbiology, Technische Universität, Braunschweig, Germany; b Centrum für Biotechnology (CeBiTec), Universität Bielefeld, Bielefeld, Germany

## Abstract

Pseudomonas aeruginosa is a frequent agent of complicated catheter-associated urinary tract infections (CAUTIs). Here, we present the improved 7.1-Mb draft genome sequence of P. aeruginosa MH19, which was isolated from a patient with an acute hospital-acquired CAUTI. It includes unique genes not represented in other P. aeruginosa genomes.

## GENOME ANNOUNCEMENT

Community-acquired urinary tract infections (UTIs) are the most prevalent kind of UTI. About 90% of these infections occur in association with urethral catheters (1). Pseudomonas aeruginosa is a pathogen frequently associated with complicated UTIs and is responsible for 35% of all catheter-associated urinary tract infections (CAUTIs) (2). The enormous adaptability and production of various virulence factors of P. aeruginosa often cause a serious course of UTIs (3, 4). In order to obtain its draft genome sequence, we extracted genomic DNA from P. aeruginosa MH19, which had been isolated from an acute hospital-acquired CAUTI (5). An 8-kb paired-end DNA fragment library was constructed for shotgun sequencing on the Genome Sequencer FLX (GS FLX) system using Titanium technology (Roche), as described recently (6, 7). Standard protocols were applied, as per the provider’s instructions. Assembly with the GS *de novo* Assembler software (Newbler) covered 216,117,934 bases from 963,947 aligned individual reads, with 215,575 paired-end reads among them. The assembled paired-end fragments had an average size of 8.7 ± 2.2 kb. Automated assembly resulted in 98 chromosomal contigs, which were organized in 5 scaffolds. The assembly data indicated the presence of a plasmid, which was experimentally confirmed by Eckhardt gel analysis (8, 9). An *in silico* gap closure approach (10, 11) was used, which reduced the chromosomal contigs to 32 while the plasmid was completed. The chromosomal scaffolds consist of 7,124,061 bp, and the plasmid-related contig has a size of 41,240 bp. The genome has a G+C content of 65.95% and is covered with an average of 30.3× by shotgun reads. The G+C content of the plasmid is 58.93%. Automated genome annotation was carried out using the GenDB software (12). This resulted in the prediction of 6,387 protein-coding sequences (CDSs) for the chromosome plus 43 CDSs for the plasmid. Four copies of the rRNA operons were identified, and 67 tRNAs were predicted. The genome sequence was compared with the core genome of P. aeruginosa using the software EDGAR (13). Thereby, unique CDSs were identified in the MH19 genome. Among these, 23 transposon-related and phage-associated genes were found that clustered in two genomic regions. Moreover, we identified heavy-metal resistance genes at two distinct genomic loci encoding a mercuric reductase, MerA (PAMH19_3342); a corresponding regulator, MerR (PAMH19_3340); a metal transport protein (PAMH19_6164) colocated with the regulatory gene *cadR* (PAMH19_6165); and a lead-cadmium transporter, CadA (PAMH19_6073). In addition, the aminoglycoside resistance gene *aadA* (PAMH19_6401) contributed to the resistance pattern of P. aeruginosa MH19. An enhanced virulence of MH19 was mirrored by a pyocin gene (PAMH19_1526). Genes homologous to the zona occludens toxin gene from Vibrio cholerae (14) (PAMH19_3943) and to a filamentous hemagglutinin gene from Bordetella pertussis (15) (PAMH19_5931) indicated horizontal gene transfer having contributed to P. aeruginosa genome evolution.

### Nucleotide sequence accession numbers.

This whole-genome shotgun project has been deposited in DDBJ/EMBL/GenBank under the accession numbers CDAZ01000001 (chromosome) and LN809998 (plasmid). The versions described in this paper are the first versions, CDAZ01000001.1 and LN809998.1, respectively.
